# Rapidly destructive osteonecrosis of the humeral head after arthroscopic rotator cuff repair: a case report

**DOI:** 10.1186/s12891-022-05494-4

**Published:** 2022-06-04

**Authors:** Myung-Seo Kim

**Affiliations:** Shoulder & Elbow Clinic, Department of Orthopaedic Surgery, College of Medicine, Kyung Hee University Hospital at Gangdong, 892, Dongnam-ro, Gangdong-gu, 05278 Seoul, Republic of Korea

**Keywords:** Anterior humeral circumflex artery, Intertubular groove, Anterolateral and intraosseous branch, Case report

## Abstract

**Background:**

Cases of rapidly destructive osteonecrosis (RDON) of the humeral head after arthroscopic rotator cuff repair (RCR) have rarely been reported, which has prevented a clear consensus on the cause of osteonecrosis.

**Case presentation:**

A 63-year-old woman without a history of trauma underwent arthroscopic RCR after being diagnosed with a medium-sized full-thickness rotator cuff tear for symptoms of left shoulder pain for six months. The patient had no medical history other than hypertension, and no other potential cause was found for osteonecrosis of the left shoulder prior to surgery. Four months after surgery, pain and range of motion improved. Six months after surgery, the patient complained of an increase in shoulder pain. While follow-up ultrasonography did not show a re-tear of the repaired tendon, osteonecrosis of the humeral head could not be confirmed as plain radiography was not performed. Follow up MRI performed a year after surgery revealed RDON of the humeral head. Despite mild improvement in the shoulder pain, the Shoulder Rating Scale of the University of California at Los Angeles (UCLA) and Constant score were poor at 23 and 69, respectively. In the present case, the arthroscopic RCR was performed using two anchors; for the repair of the anterior of the supraspinatus and the rotator interval, a 2.8-mm all-suture anchor was inserted into the upper part of the intertubercular groove. The cause of RDON is presumed to be the damage to the anterolateral and intraosseous branches of the anterior humeral circumflex artery (AHCA) for anchor positioning and insertion.

**Conclusions:**

A poor outcome was obtained in the case of RDON, despite the integrity of the repaired rotator cuff tendon after arthroscopic RCR was intact. Although the cause of RDON has not been clearly established, care should be taken not to damage the anterolateral and intraosseous branches of the AHCA regarding the insertion location of the suture anchor, and to prepare the anchor in the vicinity of the intertubercular groove.

## Background

Rapidly destructive osteonecrosis (RDON) of the humeral head is a well-known complication that has been reported to occur due to trauma, use of corticosteroids, autosomal recessive disorders such as sickle cell anemia, alcohol and dysbarism [1, 2]. However, RDON occurs very rarely after arthroscopic rotator cuff repair (RCR), while it is a severe complication leading to poor clinical outcome without postoperative recovery [3, 4]. Although the incidence of RDON has not been clearly reported after arthroscopic surgery, it has been reported that only 0.28% of patients underwent shoulder arthroplasty due to RDON after arthroscopy surgery over a period of about 9 years [4]. In addition, the cause and main demography of affected patients remain unclear owing to the lack of previous studies. A small number of studies reported that osteonecrosis of humeral head was observed in patients who underwent arthroscopic RCR using metal anchors after surgery. They suggested that a potential cause could be the multiple metal suture anchors reducing the blood supply to the anteroinferior part of the humeral head [5, 6]. Whereas, in the only multicenter case series reporting RDON after arthroscopic RCR, eight patients showed osteonecrosis within a year of surgery [3], while no patient underwent surgery using a metal anchor. In addition, there was a study that reported that RDON could occur due to damage to the anterior circumflex humeral artery (ACHA), which is responsible for blood supply of the humeral head, depending on the location of the suture anchor. However, it was a cadaver study, not a study that analyzed actual patients [7]. Thus, owing to the lack of studies on exceptionally rare cases of humeral head osteonecrosis after arthroscopic RCR, the cause is yet to be clearly identified. In this report, a potential cause of RDON is presented with respect to the position of the suture anchor in a patient who underwent arthroscopic RCR without a metal anchor.

## Case presentation

**Fig. 1:**
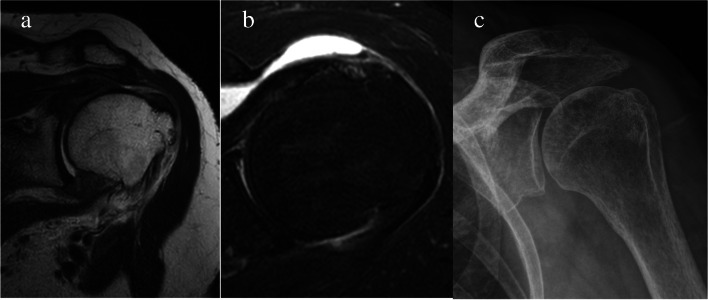
**a** A medium size rotator cuff tear was observed in the pre-operative magnetic resonance imaging and T2-weighted coronal view; **b** a nearly complete tear in the biceps long head tendon was observed in the axial view; **c** pre-operative plain radiography: No RDON was observed

A 63-year-old woman without a history of trauma presented with left shoulder pain for six months. The patient was right-hand dominant.The comorbidity was hypertension, whereas the patient had no risk factor of osteonecrosis including a dysbarism history or autoimmune disease of sickle cell anemia or systemic lupus erythematous [1, 2]. The patient had no history of smoking or alcohol consumption. The patient had a history of RCR on the right shoulder at a local clinic five years ago. Plain radiography performed at our institution did not reveal osteonecrosis on the right shoulder. Before visiting our institution, the patient had received treatments comprising nonsteroidal anti-inflammatory medication and physiotherapy, including active assisted stretching exercises in a local clinic. The patient did not receive corticosteroid medication [2]. The patient presented with left shoulder pain accompanied by stiffness in the initial physical examination performed at our institution, and additional conservative treatment, excluding steroids, was given for a 1-month period. Despite continued conservative treatment, the pain did not improve, which led to arthroscopic RCR. The inflammatory markers (white blood cell count, white blood cell differential for segmented neutrophils, erythrocyte sedimentation rate, and C-reactive protein) before surgery were within normal ranges. The visual analog scale (VAS) score of the left shoulder a day before surgery was 3 for pain at rest and 8 for pain during activity. The range of motion was 120° for active forward flexion, 30° for external rotation at the side, 40° for external rotation at 90° of abduction, 30° for internal rotation at 90° of abduction, and L3 vertebral level for internal rotation to the posterior (hand behind back), accompanied by stiffness. A medium size rotator cuff tear and nearly complete tear of the biceps long head tendon were observed on pre-operative magnetic resonance imaging (Fig. 1a, b). Osteonecrosis of the humeral head was not observed on plain radiography (Fig. 1c).

**Fig. 2:**
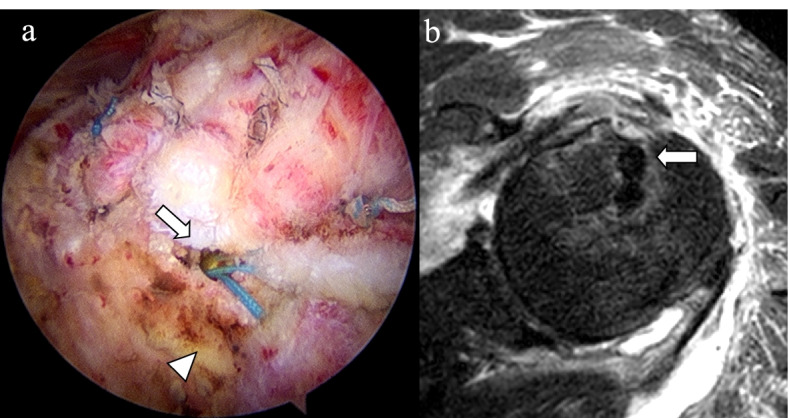
**a** Arthroscopic RCR was performed. A 2.8 mm all-suture anchor was inserted to the upper part of the intertubercular groove (arrow), and to examine the anchor settling, the area surrounding the anchor was given the debridement using radiofrequency (arrowhead); **b** the insertion of the anchor to the intertubercular groove is shown on the immediate post-operative MRI (arrow)

Arthroscopic RCR was performed under general anesthesia in the beach chair position. As the biceps long head tendon showed a nearly complete tear, tenotomy was performed using radiofrequency in the area attached to the superior labrum. For the subscapularis, which showed a longitudinal tear with a relatively intact footprint of the lesser tuberosity, only debridement was performed. Acromioplasty was performed by the standard procedure, and RCR was performed in a single row using two anchors: a 2.8 mm all-suture anchor (Y-Knot®; ConMed Linvatec, Largo, FL) and a 5.5 mm open-construct PEEK suture anchor (HEALICOIL; Smith & Nephew, Andover, MA). No metal anchor was used. For the PEEK suture anchor, the insertion was in the lateral aspect of the greater tuberosity, and after the single-row repair, an all-suture anchor was inserted into the upper part of the intertubercular groove to repair the anterior of the supraspinatus and the rotator interval. To examine anchor settling, radiofrequency debridement was performed on the surrounding area (Fig. 2a, b). The retracted torn rotator cuff showed repair up to the footprint of the greater tuberosity without tension. On the immediate postoperative follow-up MRI, osteonecrosis of the humeral head was not detected. During surgery, epinephrine was not added to the irrigation fluid, which was controlled within 50–70 mmHg using a pressure-controlled pump. After surgery, local anesthetics were not used in the intra-articular or subacromial space. The patient performed assisted motion exercises that started six weeks after surgery, when the abduction brace was weaned.

**Fig. 3:**
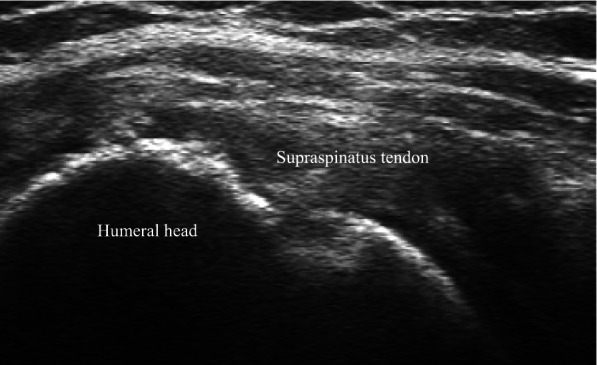
Post-operative six-month follow-up ultrasonography: the integrity of the repaired rotator cuff tendon was intact

**Fig. 4:**
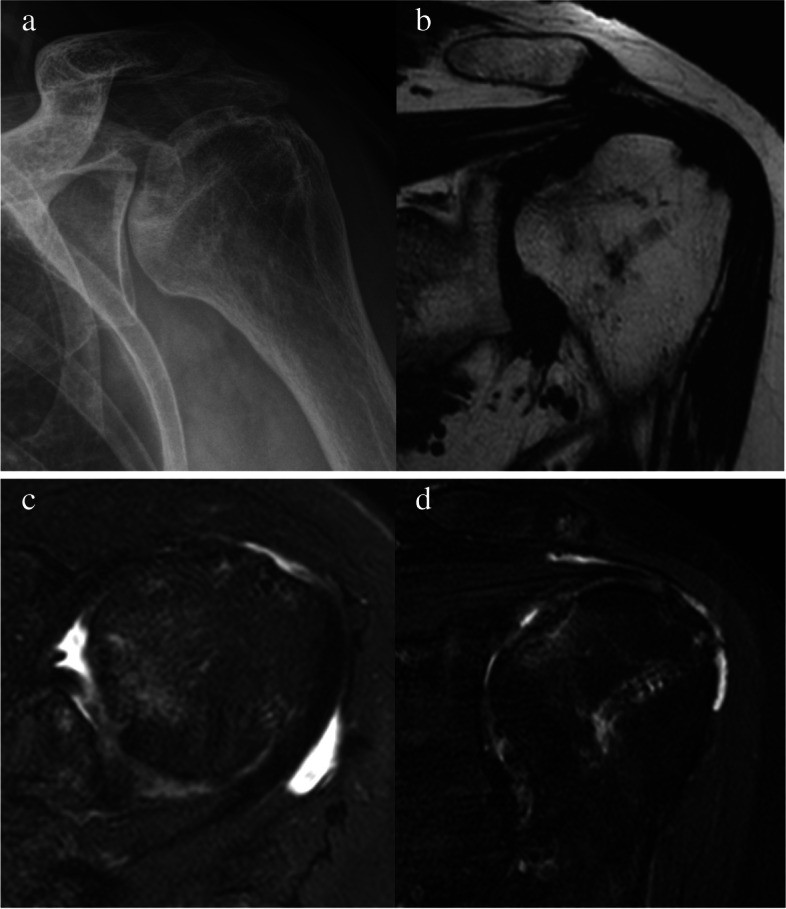
**a**, **b**, **c** Post-operative one-year follow-up plain radiography and MRI; osteonecrosis of the humeral head on the superomedial side can be seen in the T1-weighted coronal and T2-weighted axial view **d** in the T2-weighted coronal view, the repaired rotator cuff tendon shows insufficient thickness although discontinuity is not visible (Sugaya classification type III)

Four months after surgery, the pain and range of motion showed significant improvements compared to the time before surgery as follows: pain (VAS score pain at rest, 2; pain during activity, 3) and range of motion (140° for active forward flexion, 30° for external rotation at the side, 40° for external rotation at 90° of abduction, 40° for internal rotation at 90° of abduction, and L3 vertebral level for internal rotation to the posterior). Six months after surgery, the left shoulder pain (VAS score pain at rest, 5; pain during activity, 6) increased, except for external rotation at 90° of abduction, all other ranges of motion were at similar level (140° for active forward flexion, 30° for external rotation at the side, 70° for external rotation at 90° of abduction, 40° for internal rotation at 90° of abduction, and L4 vertebral level for internal rotation to the posterior). On follow-up ultrasonography, the integrity of the repaired rotator cuff tendon was intact (Fig. 3), but osteonecrosis could not be confirmed due to the lack of plain radiography. The inflammatory markers estimated to detect infection were within normal ranges. Conservative treatment including NSAIDs and stretching exercises was continued, and 1 year after surgery, the left shoulder pain decreased (VAS score pain at rest: 1, pain during activity: 3); however, follow-up plain radiography and MRI showed osteonecrosis of the humeral head (Fig. 4a, b, c). The repaired rotator cuff tendon showed insufficient thickness, but no discontinuity (Sugaya classification type III) (Fig. 4d). Shoulder range of motion was similar to the time after six months of surgery as follows: 140° for active forward flexion, 30° for external rotation at the side, 70° for external rotation at 90° of abduction, 50° for internal rotation at 90° of abduction, and L2 vertebral level for internal rotation to the posterior. The serial shoulder range of motion from before surgery to final follow-up is summarized in Table 1. The Shoulder Rating Scale of the University of California at Los Angeles (UCLA) and Constant scores were 23 and 59, respectively, indicating poor clinical outcome. The patient had gradual pain relief and arthroplasty was not performed.

**Table 1:** The serial shoulder range of motion from before surgery to final follow-up

ROM	Pre-op	4 months after surgery	6 months after surgery	1 year after surgery
aFF	120	140	140	140
ERs	30	30	30	30
ERa	40	40	70	70
IRa	30	40	40	50
IRp	L3	L3	L4	L2

*ROM* Range of motion, *aFF* Active forward flexion, *ERs* External rotation to the side, *ERa* External rotation at 90° of abduction, *IRa* Internal rotation at 90° of abduction, *IRp* Internal rotation to the posterior

## Discussion and conclusions

In this case study, RDON occurred on MRI performed 1 year after arthroscopic RCR in a 63-year old woman and poor clinical outcome was obtained. Arthroscopic RCR was performed using two suture anchors, among which a 2.8-mm small diameter all-suture anchor was inserted into the upper part of the intertubercular groove. The placement of the suture anchor and the use of radiofrequency around the anchor location may cause damage to the AHCA, which is responsible for the blood supply of the humeral head.

A remarkable increase in the incidence rate of RCR has been reported [8] with a consequent diversity of complications [9]. RDON of the humeral head is a complication that rarely occurs after arthroscopic RCR, for which additional surgery is required due to poor prognosis in most cases [4]. Therefore, surgeons should not overlook these significant complications. Dilisio et al. reported that reverse total shoulder arthroplasty was performed for severe pain, poor shoulder function, and glenohumeral arthritis in all patients with RDON after arthroscopic surgery [4].

Nevertheless, only a few case reports have been published until recently, and these reports have suggested varying potential causes. Thus, there is no consensus on the cause of RDON of the humeral head that may occur after arthroscopic RCR. Beauthier et al*.* reported osteonecrosis of the humeral head eight months after arthroscopic RCR was performed using three metal anchors on the greater tuberosity. They presumed that the use of multiple metal anchors could have increased the pressure on the humeral head to obstruct the blood flow [5]. Goto et al*.*, in contrast, reported RDON after 3 months of arthroscopic RCR despite the use of two metal anchors in the insertion with a sufficient anterior–posterior interval. Thus, these two studies have reported contrasting results.

In addition, Goto et al. suggested the use of a metal anchor as a potential cause of RDON and damage to the vasa vasorum of the humeral head caused by the insertion of multiple anchors [6]. In contrast, in a multicenter study by Kim et al. on eight patients for whom no metal anchor was used, the RDON was reported within one year of arthroscopic RCR [3]. Similarly, in this case report, RDON occurred in a patient who received arthroscopic RCR using an all-suture anchor and one PEEK suture anchor. The findings of the previous studies and this case report collectively indicate a low probability of a metal anchor being the primary cause of RDON.

In previous studies, most patients with RDON after arthroscopic RCR were older and female, with RDON on the right-dominant side [3, 5, 6]. Kim et al*.* reported RDON within 1 year of arthroscopic RCR in eight patients, which was the greatest sample size in a single study; the patients were all women, with a mean age of 64 years, and RDON was located on the right-dominant side. All patients included in their study were right-hand dominant.

In this study, RDON occurred after arthroscopic RCR in a 63-year-old woman, although it was not observed on the dominant side.

To prevent RDON, Keough et al*.* highlighted the importance of avoiding damage to the vascularization of the humeral head upon arthroscopic RCR, while care should be taken not to damage the ACHA and anterolateral branch [7]. The vascular supply to the humeral head mainly involves the ACHA and posterior circumflex humeral artery [10, 11]. The anterolateral branch of the ACHA is the main vessel that runs along the lateral side of the biceps long head tendon with a course superior to the intertubercular groove [5]. Keough et al*.* reported that the superomedial aspect of the humeral head was a high-risk zone for osteonecrosis with a lack of arterial perfusion and that damage to the intraosseous arterial network of ACHA during arthroscopic RCR could cause RDON in the high-risk zone [11]. In this case report, the insertion of a suture anchor was performed on the upper part of the intertubercular groove to repair the anterior supraspinatus tendon and the rotator interval. In addition, to examine anchor settling, the area surrounding the insertion was debrided using radiofrequency, presumed to have damaged the anterolateral and intraosseous branches of the AHCA. Unfortunately, information regarding anchor locations was not available in previously reported case reports. Thus, its causative role in the development of RDON remains uncertain.

In this study, the definite cause of RDON could not be concluded, as only a single case is reported. Nevertheless, in the present case, RDON occurred despite the use of a relatively small all-suture anchor of 2.8 mm diameter. It is thus presumed that the area of anchor insertion and the damage to the network of ACHA branches had a significant influence on the incidence of RDON. Thus, care should be taken with respect to the location of anchor insertion to preserve the blood supply to the humeral head upon arthroscopic RCR, especially in elderly female patients. The use of radiofrequency also requires attention during anchor insertion into the upper part of the intertubular groove.

## Data Availability

The authors declare that all data used in the study appear in the submitted article.

## Abbreviations

RCR: Rotator cuff repair
RDON: Rapidly destructive osteonecrosis
MRI: Magnetic resonance imaging
VAS: Visual analogue scale
UCLA: University of California at Los Angeles
AHCA: Anterior humeral circumflex artery

## References

[1] Hernigou P, Hernigou J, Scarlat M (2020). Shoulder Osteonecrosis: Pathogenesis, Causes, Clinical Evaluation, Imaging, and Classification. Orthop Surg.

[2] Harreld KL, Marker DR, Wiesler ER, Shafiq B, Mont MA (2009). Osteonecrosis of the humeral head. J Am Acad Orthop Surg.

[3] Kim JK, Jeong HJ, Shin SJ, Yoo JC, Rhie TY, Park KJ, Oh JH (2018). Rapid Progressive Osteonecrosis of the Humeral Head After Arthroscopic Rotator Cuff Surgery. Arthroscopy.

[4] Dilisio MF, Noble JS, Bell RH, Noel CR (2013). Postarthroscopic humeral head osteonecrosis treated with reverse total shoulder arthroplasty. Orthopedics.

[5] Beauthier V, Sanghavi S, Roulot E, Hardy P (2010). Humeral head osteonecrosis following arthroscopic rotator cuff repair. Knee Surg Sports Traumatol Arthrosc.

[6] Goto M, Gotoh M, Mitsui Y, Okawa T, Higuchi F, Nagata K (2015). Rapid collapse of the humeral head after arthroscopic rotator cuff repair. Knee Surg Sports Traumatol Arthrosc.

[7] Keough N, Lorke DE (2021). The humeral head: A review of the blood supply and possible link to osteonecrosis following rotator cuff repair. J Anat.

[8] Paloneva J, Lepola V, Aarimaa V, Joukainen A, Ylinen J, Mattila VM (2015). Increasing incidence of rotator cuff repairs–A nationwide registry study in Finland. BMC Musculoskelet Disord.

[9] Desai VS, Southam BR, Grawe B (2018). Complications Following Arthroscopic Rotator Cuff Repair and Reconstruction. JBJS Rev.

[10] Gerber C, Schneeberger AG, Vinh TS (1990). The arterial vascularization of the humeral head. An anatomical study. J Bone Joint Surg Am.

[11] Keough N, de Beer T, Uys A, Hohmann E (2019). An anatomical investigation into the blood supply of the proximal humerus: surgical considerations for rotator cuff repair. JSES Open Access.

